# Potential applicability of the importation risk index for predicting the risk of rarely imported infectious diseases

**DOI:** 10.1186/s12889-023-16380-6

**Published:** 2023-09-12

**Authors:** Kyung-Duk Min, Sun-Young Kim, Yoon Young Cho, Seyoung Kim, Joon-Sup Yeom

**Affiliations:** 1 College of Veterinary Medicine, Chungbuk National University, 1 Chungdae-ro, Seowon-gu, Cheongju, 28644 South Korea; 2 Department of Public Health Sciences, Graduate School of Public Health, Seoul National University, 1 Gwanak-ro, Gwanak-gu, Seoul, 08826 South Korea; 3 Institute of Health and Environment, Graduate School of Public Health, Seoul National University, 1 Gwanak-ro, Gwanak-gu, Seoul, 08826 South Korea; 4 Division of Infectious Disease, Department of Internal Medicine, Yonsei University College of Medicine, 50-1 Yonsei-ro, Seodaemun-gu, Seoul, 03722 South Korea

**Keywords:** Rabies, Sleeping sickness, Disease importation

## Abstract

**Background:**

There have been many prediction studies for imported infectious diseases, employing air-travel volume or the importation risk (IR) index, which is the product of travel-volume and disease burden in the source countries, as major predictors. However, there is a lack of studies validating the predictability of the variables especially for infectious diseases that have rarely been reported. In this study, we analyzed the prediction performance of the IR index and air-travel volume to predict disease importation.

**Methods:**

Rabies and African trypanosomiasis were used as target diseases. The list of rabies and African trypanosomiasis importation events, annual air-travel volume between two specific countries, and incidence of rabies and African trypanosomiasis in the source countries were obtained from various databases.

**Results:**

Logistic regression analysis showed that IR index was significantly associated with rabies importation risk (p value < 0.001), but the association with African trypanosomiasis was not significant (p value = 0.923). The univariable logistic regression models showed reasonable prediction performance for rabies (area under curve for Receiver operating characteristic [AUC] = 0.734) but poor performance for African trypanosomiasis (AUC = 0.641).

**Conclusions:**

Our study found that the IR index cannot be generally applicable for predicting rare importation events. However, it showed the potential utility of the IR index by suggesting acceptable performance in rabies models. Further studies are recommended to explore the generalizability of the IR index’s applicability and to propose disease-specific prediction models.

**Supplementary Information:**

The online version contains supplementary material available at 10.1186/s12889-023-16380-6.

## Introduction

Disease importation and exportation have become significant global health issues due to the expansion of international travels. Recent pandemics, such as coronavirus disease 2019 (COVID-19) and the influenza in 2009, have demonstrated the tremendous impact that a local outbreak can have on global society. Not only pandemics but several tropical diseases including dengue and malaria have continuously been reported in non-endemic areas due to international travel. In South Korea, for example, the number of imported dengue cases increased from 13 in 2003 to 149 in 2012 [1]. To minimize the impact of disease importation, various studies have been conducted to predict the disease importation risks, focusing on human-to-human transmission infectious diseases, such as COVID-19 [2] and measles [3], or vector-borne diseases [4], including chikungunya and malaria. These importation risk assessments have proven highly applicable to the development of effective quarantine policies, as they improve vigilance and strengthen the capacity to respond to the imported cases [5].

Studies estimating the importation risk often rely on international travel volume as the primary predictor. For example, Gardner and Sarkar [6] estimated risk of dengue importation for each airport worldwide using air travel volume, local population size and interpolated suitability of vector mosquitos. Russell et al. [7] also estimated the number of imported COVID-19 cases by using the product of the prevalence in source countries and travel volumes. Quam and Wilder-Smith [8] suggested a simple indicator of disease importation risk (IR), which can be calculated by multiplying travel volume and reported incidence in source countries. However, there have been insufficient quantitative studies examining the validity of using travel volume or IR index to predict disease importation risk. Although several studies have analyzed the validity of travel volume as the main predictor [9, 10], their focus has been on diseases that are frequently imported. However, it remains to be questioned whether the IR index can be effectively utilized to predict the importation risk of rare diseases that are not reported annually and only have a small number of cases per year. The predictions for rare diseases, in contrast to those for frequently reported diseases, are challenging due to limited data availability. Nevertheless, there is a substantial demand for reliable predictions, especially in the case of highly fatal diseases, such as Ebola, which can cause significant societal disruptions upon secondary transmission in the importing country.

In this study, we aimed to investigate whether IR index or travel volume can be used as predictors for the risk of disease importation that have rarely been reported. To this end, the association of disease importation risks with IR index or travel volume were examined using multi-national datasets. Rabies and African trypanosomiasis were chosen as target diseases because the data on the incidence rates of these diseases were available, and disease importation case reports were more sensitive due to their high case-fatality rate.

## Methods

### Study framework and data acquisition

As our primary candidate for the predictor, we examined the association between the risk of disease importation and the IR index using data from various sources and imported countries. We calculated predictability of the IR index. In the case of a poor prediction performance by the IR index, we developed an alternative prediction model using travel volume and the nominal variable of source countries as predictors.

In the analysis, we only included countries that have reported at least one imported or exported cases of rabies or African trypanosomiasis between 2010 and 2019 (10 years). By excluding countries with no reports of imported cases, we aimed to minimize potential bias arising from inclusion of countries that possibly have experienced the diseases importation but did not report it due to a lack of surveillance capacity. To acquire the list of diseases imported, we used reports from ProMed-Mail [11], the GIDEON database [12], and published literature. ProMed-Mail is a web-based archive that includes disease outbreak reports, providing information on disease importation events. These events can be searched using specific search termssuch as “rabies ex” and “African trypanosomiasis ex” for rabies and African trypanosomiasis, respectively, where “ex” denotes exportation. GIDEON database also provides various disease outbreak records including importation events with references. We utilized the search function in the database to complie the list of the importation events.

Because our analysis included only countries with at least one imported or exported case between 2010 and 2019, the units of analysis for this study are the pairs of included countries for each year. For example, if there is three imported countries and four exported countries (source countries), the number of units in this study is 120, because the possible pairs between source countries and imported countries are 12 (= 3*4) and the pairs are replicated each year (10 years). The response variable is defined as whether there is at least one importation event. For instance, if there is disease importation event between country A and B only in 2012, the study unit representing the pair of countries A and B in 2012 is categorized as an event unit. On the other hand, the study units representing the pairs of countries A and B in other years are categorized as non-events units.

IR index was calculated for each pair of source and imported countries, as well as for each year in the form of the product of yearly travel volume and the annual incidence of source countries. The annual number of passengers by air travels was obtained from OAG, an air travel intelligence company specialized in airline industry data [13]. OAG not only provided the total number of passengers, but also stratified the numbers by airline seat class (i.e., classified into first class, business class and economy class). The annual incidence data of source countries were acquired from the Institute for Health Metrics and Evaluation (IHME) database [14]. The IHME database offers a comprehensive range of global health statistics including burden of diseases.

### Statistical analysis

Descriptive analysis for IR index were conducted to explore temporal or geographical trends of the variable. We calculated and analyzed the average IR indices for both target diseases by each imported country and each year. In addition, average IR indices between two specific countries were analyzed, categorizing them by geographic regions and income-level.

Logistic regression models were employed to examine the association between the candidate predictors and disease importation risk, as well as to assess predictability of the variables. To determine the validity of the predictors, we considered the results to meet the following criteria: (1) the coefficient in the logistic regression model was significantly positive (coefficients of the explanatory variable and corresponding p value were presented to this end), (2) Hosmer and Lemeshow goodness of fit (HL GOF) test was not significant (p value > 0.05), and (3) area under curve for Receiver operating characteristic (AUC) was higher than 0.7, which is generally considered an acceptable cut-off value [15]. As the main goal of this study is to evaluate the prediction performance of the IR indices, we split the dataset into two parts: a training set and a test set. The data from 2010 to 2015 (6 years) were used for the training set, while the data from 2016 to 2019 (4 years) were used for the test set. The prediction models were developed using only the training set, and the AUC was estimated using only the test set. Because we employed univariable models, potential confounding factors were not included as covariates. We suggested coefficients and p value from the models to show the direction and statistical significance of the associations. Considering that residuals from the logistic regression models could be correlated by source countries, we also conducted generalized estimating equation (GEE) models with exchangeable correlation structures. Coefficients and p-values from the GEE models were also suggested.

## Results

The lists of disease importation cases and their references are shown in the Supplementary material. In total, 23 rabies and 23 African trypanosomiasis cases were identified. For rabies, importation events were reported every year from 2010 to 2019,  with the highest number of imported events (5 cases) was recorded in 2012. Within the study period, fifteen countries have reported imported rabies events and the United States showed the highest number of events (3 cases). Fourteen countries have reported exported rabies events, with India having the highest number of events (14 cases). Regarding African trypanosomiasis, there were no imported events in 2011, and the number of the imported events was highest in 2012 and 2017 each with four cases. Within the study period, eleven countries have reported the imported events, and South Africa showed the highest number of events (6 cases). As for exported events, six countries reported, with Zambia having the highest number of events (10 cases).

**Fig. 1:**
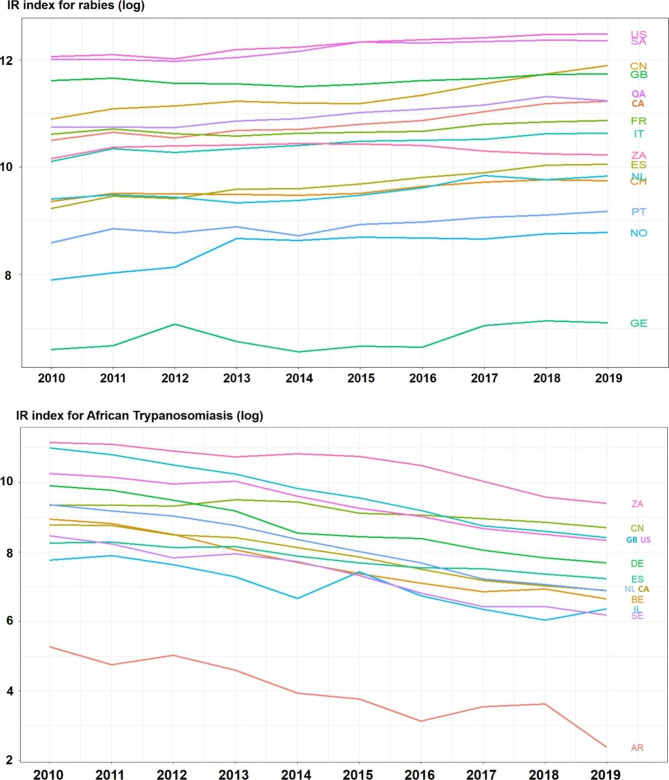
Time trend of average IR indices for rabies and African trypanosomiasis Note: AR = Argentina, CA = Canada, CH = Switzerland, CN = China, DE = Germany, ES = Spain, FR = France, GB = United Kingdom, GE = Georgia, IL = Israel, IT = Italy, NL = Netherlands, NO = Norway, PT = Portugal, QA = Qatar, SA = Saudi Arabia, SE = Sweden, US = United States, ZA = South Africa

**Fig. 2:**
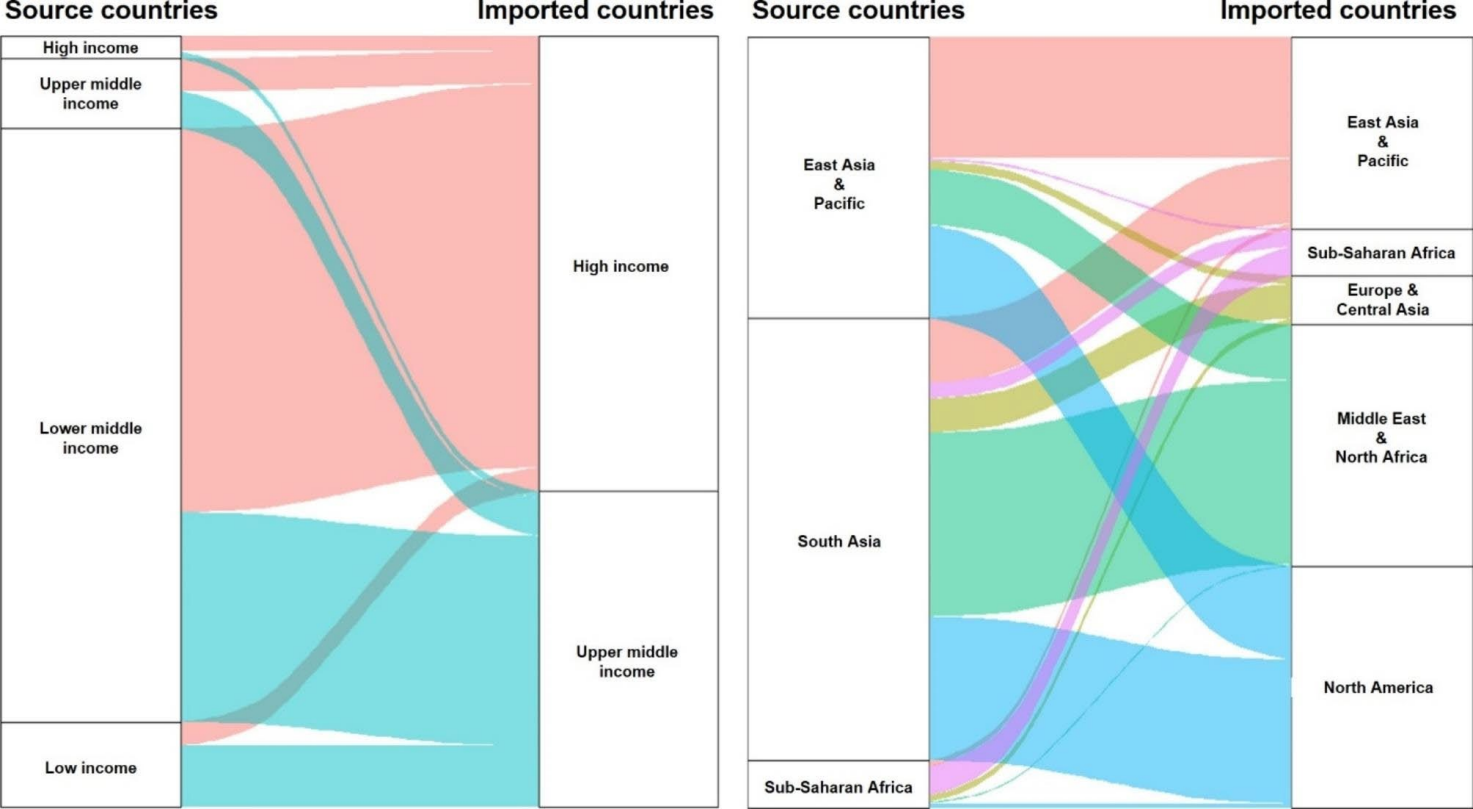
Comparison of IR index for rabies between source and imported countries categorizing by income-levels and geographical regions Note: thicker stream indicates higher IR index for given categories of source and imported countries

**Fig. 3:**
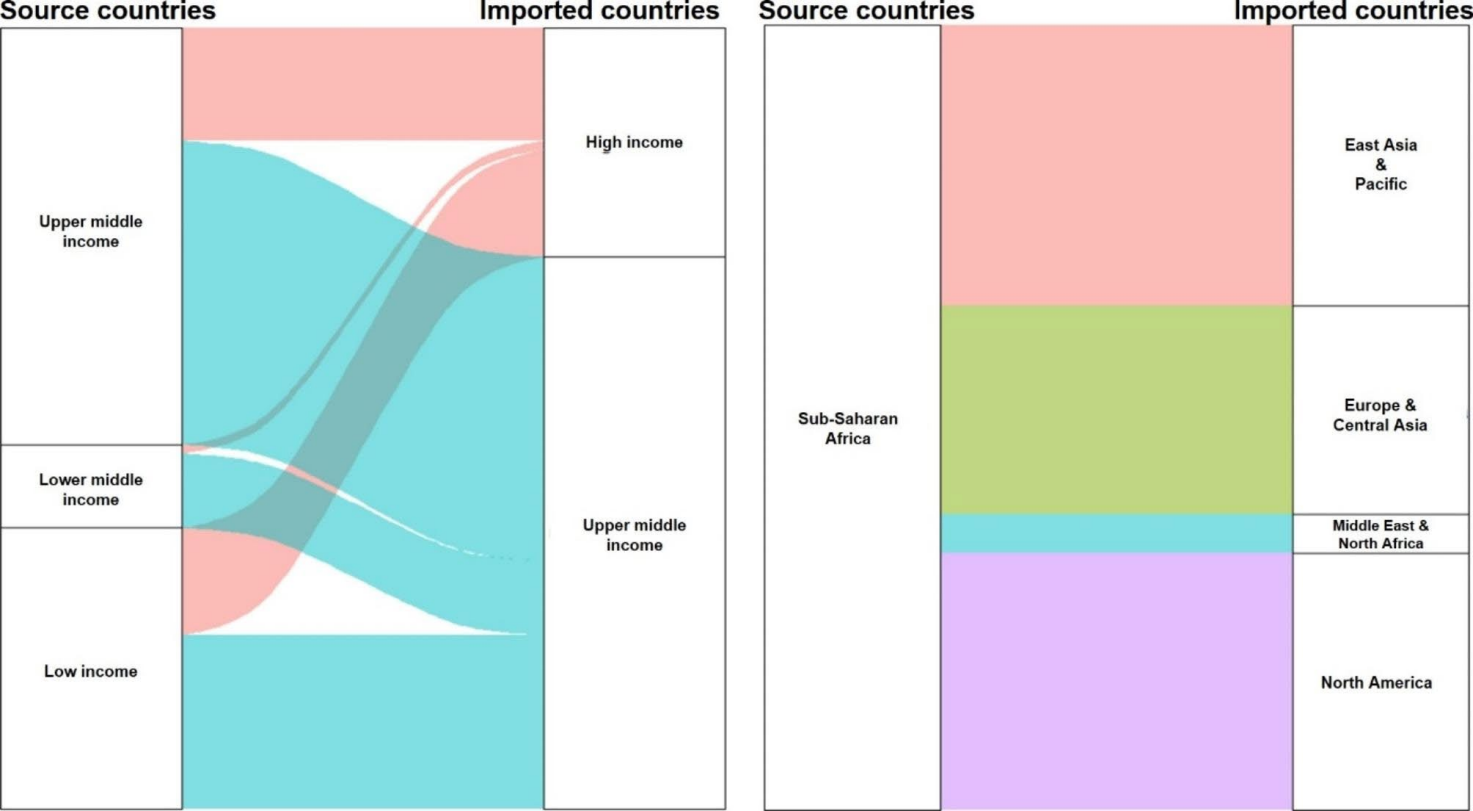
Comparison of IR index for African trypanosomiasis between source and imported countries categorizing by income-levels and geographical regions Note: thicker stream indicates higher IR index for given categories of source and imported countries

Figure 1 showed the temporal trend of average IR indices for both rabies and African trypanosomiasis by imported countries. The IR index for rabies tends to slightly increase but that for African trypanosomiasis tends to decrease, generally. Comparisons of the average IR indices between geographic regions and countries’ income-level were illustrated in Figs. 2 and 3, respectively. For rabies, the IR index is higher in lower middle-income countries and South Asian countries than other source countries. On the other hand, the IR index for African trypanosomiasis is higher in upper middle-income countries than oher source countries.

Table 1 showed the results of the logistic regression analysis that examined the validity of the IR index as a predictor for rabies importation events. Irrespective of the airline seat class, the association of the rabies importation risk with the number of passengers via air-travel was significantly positive. However, the goodness-of-fit test for the model using the number of business class passengers showed a lack of fit to the data. All models showed acceptable performance in classifying whether there are imported events, as their AUC values were close to or higher than 0.7. The model fitted using economy class of seats showed the highest performance. Both GLM and GEE models showed similar results indicating robustness of the findings.

**Table 1:** Assessment for the validity of importation risk index as a predictor for Rabies importation by logistic regression (GLM) and generalized estimating equation (GEE) analysis

Model	Type of travel-volume	GLM			GEE		
		Coef^1^	*p* value^1^	AUC^2^	Coef^1^	*p* value^1^	AUC^2^
Rabies Model 1	Total	0.133	< 0.001	0.728	0.133	< 0.001	0.728
Rabies Model 2	1st class	5.748	< 0.001	0.682	6.289	< 0.001	0.682
Rabies Model 3	2nd class	4.251	< 0.001	0.689	4.623	< 0.001	0.689
Rabies Model 4	Economy class	0.141	< 0.001	0.734	0.140	< 0.001	0.734

*Note*: The response variable is whether there is at least one imported case between two specific countries in a specific year. The importation risk index (IR index) was defined as product of travel-volume and disease burden in the source countries. As there were four variables for the travel-volume (number of 1st class passengers, number of 2nd class passengers, number of economy class passenger and total number of all passengers), four IR indices can be calculated. Four logistic regression models and four GEE models were fitted using the four IR indices, respectively as the index was the only independent variable in each model. The IR index variable in these models were rescaled by dividing 10^5 to increase coefficient. *P* values of Hosmer and Lemeshow goodness of fit in the GLM models were 0.429, 0.504, 0.038, and 0.348 for the model 1–4, respectively, indicating lack of fit for the Rabies Model 3

^1^coefficients and *p* values for importation risk index

^2^area under curve for Receiver operating characteristic

An equivalent analysis for African trypanosomiasis, however, showed that the association between the importation event risk and IR index was not significant, and their prediction performance in categorizing whether there is an event of the importation was poor (AUC < 0.5) (Table 2). In alternative models using travel volume and the nominal variable of each source country (Table 3), the AUC was close to or higher than 0.5, implying unacceptable predictability. The coefficients for travel volume were significantly positive in the model using the total, 2nd, and economy class passengers.

**Table 2:** Assessment for the validity of importation risk index as a predictor for African trypanosomiasis importation by logistic regression (GLM) and generalized estimating equation (GEE) analysis

Model	Type of travel-volume	GLM			GEE		
		Coef^1^	*p* value^1^	AUC^2^	Coef^1^	*p* value^1^	AUC^2^
AT Model 1	Total	-0.092	0.923	0.319	-0.590	0.590	0.319
AT Model 2	1st class	-123.541	0.244	0.457	-141.454	0.360	0.457
AT Model 3	2nd class	-8.622	0.629	0.329	-16.912	0.550	0.329
AT Model 4	Economy class	-0.055	0.958	0.313	-0.559	0.630	0.313

*Note*: The response variable is whether there is at least one imported case between two specific countries in a specific year. The importation risk index (IR index) was defined as product of travel-volume and disease burden in the source countries. As there were four variables for the travel-volume (number of 1st class passengers, number of 2nd class passengers, number of economy class passenger and total number of all passengers), four IR indices can be calculated. Four logistic regression models and four GEE models were fitted using the four IR indices, respectively as the index was the only independent variable in each model. The IR index variable in these models were rescaled by dividing 10^5 to increase coefficient. *P* values of Hosmer and Lemeshow goodness of fit in the GLM models were 0.423, 0.583, 0.462, and 0.078 for the model 1–4, respectively, indicating lack of fit for the AT Model 4

^1^coefficients and *p* values for importation risk index

^2^area under curve for Receiver operating characteristic

**Table 3:** Assessment for the validity of travel volume as a predictor for African trypanosomiasis importation by logistic regression (GLM) and generalized estimating equation (GEE) analysis

Model	Type of travel-volume	GLM			GEE		
		Coef^1^	*p* value^1^	AUC^2^	Coef^1^	*p* value^1^	AUC^2^
AT Model 5	Total	0.341	0.032	0.640	0.411	< 0.001	0.542
AT Model 6	1st class	-15.098	0.498	0.492	-21.408	0.340	0.523
AT Model 7	2nd class	8.608	0.082	0.505	10.840	< 0.001	0.547
AT Model 8	Economy class	0.375	0.030	0.641	0.450	< 0.001	0.542

*Note*: The response variable is whether there is at least one imported case between two specific countries in a specific year. Independent variables were travel-volume and nominal variable of source countries. As there were four variables for the travel-volume (number of 1st class passengers, number of 2nd class passengers, number of economy class passenger and total number of all passengers), four logistic regression models and four GEE models were fitted using the different travel-volume variables, respectively. The travel-volume variable in these models were rescaled by dividing 10^5 to increase coefficient. *P* values of Hosmer and Lemeshow goodness of fit in the GLM models were 0.539, 0.667, 0.447, and 0.540 for the model 5–6, respectively, indicating there is no lack of fit for the AT Models

^1^coefficients and *p* values for travel volume

^2^area under curve for Receiver operating characteristic

## Discussion

We aimed to investigate whether the IR index or travel volume could be used to predict the risk of disease importation in countries with rare reports of imported cases. To this end, we examined the predictability of IR index and travel volume, as candidate predictors for disease importation risk, using multi-national data. Rabies and African trypanosomiasis were chosen as target diseases. Our results revealed that IR index can be used as a predictor for rabies importation. However, we found that IR index was not applicable for the prediction of African trypanosomiasis importation.

Our results suggested that IR index cannot be generally used as a predictor for the importation risk of rare diseases such as the African trypanosomiasis, despite its conceptual plausibility. There are two possible explanations for the poor performance in predicting African trypanosomiasis importation risk. First, incidence rate of a disease may not represent the true risk of infection in the source countries. For instance, people in a source country tend to have immunity against African trypanosomiasis due to the endemic nature of the disease [16], and thus, the incidence rate in the country may not reflect the true risk of infection for travelers in the country. Therefore, infection risk of international travelers cannot be estimated solely based on the incidence among the local population. Second, sensitivity of surveillance in different countries would differ, leading to incompatibility of incidence rate measures among different source countries. Additionally, we found that the association between importation risk and travel volume can vary by the types of airline seat class. The finding suggested that economy class travelers have a higher risk of infection in the source countries than the first class or business class travelers. The results were consistent with a previous study by Furuya-Kanamori et al. [17], which found that backpackers usually have a higher risk of local infection than other types of travelers (e.g., business trips) in terms of travel purposes.

The study results suggest that the IR index cannot be generally applicable for predicting rare importation events. However, they also show the potential applicability of the IR index by demonstrating acceptable performance in rabies models. Further studies are recommended to explore the generalizability of the IR index’s applicability by targeting other diseases and to propose disease-specific prediction models.

There are several limitations in this study. First, we used a dichotomous outcome variable (that is, whether there are disease importation events or not) rather than counting the number of imported cases for each event. Second, IHME data was used as a measure of disease incidence in the source countries. Considering that the incidence rates from the IHME database are estimates based on data from diverse sources and assumptions, alternative ways of estimating disease incidence may yield different estimated risks using various prediction models. Third, there could be a selection bias in our data as we deliberately excluded countries with no reports of imported cases. Fourth, the uncertainty of the estimated predictability from IR index should be considered. Since we used only one variable in the models, the effect of including other variables (e.g., socioeconomic status of source/imported countries) was ignored, which could potentially impact the strength of the variable. Finally, quarantine-related policies for the travelers (e.g., pre-travel consultation or vaccination against rabies before travels) can be different by countries, but we did not take those factors into consideration due to lack of accessible data.

## Conclusion

In this study, we investigated whether the IR index or travel volume can be used to predict the risk of disease importation in the countries with rare reports of imported cases. Our findings suggested the potential utility of the IR index to estimate disease importation risks even in regions where imported cases were rarely reported. However, it is important to note that the performance of IR index was different between the the two target diseases.  Therefore, further studies are highly necessary to confirm whether the IR index or travel volume can be effectively used for other types of diseases, especially those with high case fatality rate, as such diseases are usually sensitive to detection. Moreover, considering that the temporal unit of our study was a year, future studies with shorter time units could be valuable to examine the predictability of IR index and travel volume.

### Electronic supplementary material

Below is the link to the electronic supplementary material.

Supplementary Material 1

## Data Availability

The importation events of rabies and African trypanosomiasis can be obtained by ProMed-Mail (https://promedmail.org/) and literature review. Supplementary materials in this study also provide a list of the importation events of the diseases. We purchased international air travel volume between countries from OAG (https://www.oag.com/) which is not publicly available. The datasets used and/or analysed during the current study are available from the corresponding author (KIM SY) on reasonable request.
