# Supplementary material for: Potential applicability of the importation risk index for predicting the risk of rarely imported infectious diseases
Source: BMC Public Health. 2023 Sep 12;23:1776. doi: 10.1186/s12889-023-16380-6 (PMC10496286; doi:10.1186/s12889-023-16380-6)
Supplement: Supplementary file 1 — Supplementary Material 1 [file 12889_2023_16380_MOESM1_ESM.docx]

Table S1. List of rabies imported events included in this study (from 2010 to 2019)

| ID | Year | Source country | Imported country | Reference^1^ |
| --- | --- | --- | --- | --- |
| 1 | 2010 | Azerbaijan | Georgia | 20101222.4507000 |
| 2 | 2011 | India | Italy | 20120206.1034574 |
| 3 | 2011 | Haiti | Netherlands | 20130625.1791201 |
| 4 | 2011 | Guinea-Bissau | Portugal | Carrara et al 2013 |
| 5 | 2012 | Dominican Republic | Canada | 20120417.1104531 |
| 6 | 2012 | United States | Switzerland | 20120829.1270822 |
| 7 | 2012 | India | China | Carrara et al 2013 |
| 8 | 2012 | India | United Kingdom | 20120523.1142429 |
| 9 | 2012 | Mozambique | South Africa | 20120515.1134088 |
| 10 | 2013 | Haiti | United States | 20110722.2208000 |
| 11 | 2014 | Indonesia | China | Gautret et al, 2020 |
| 12 | 2014 | Morocco | Spain | Gautret et al, 2020 |
| 13 | 2014 | Mali | France | 20140404.2381608 |
| 14 | 2014 | India | Qatar | Gautret et al, 2020 |
| 15 | 2015 | Bangladesh | France | Gautret et al, 2020 |
| 16 | 2015 | Philippines | United States | Gautret et al, 2020 |
| 17 | 2016 | Morocco | Saudi Arabia | Gautret et al, 2020 |
| 18 | 2017 | Sri Lanka | France | Gautret et al, 2020 |
| 19 | 2017 | India | United States | Gautret et al, 2020 |
| 20 | 2018 | Morocco | United Kingdom | 20181113.6142425 |
| 21 | 2019 | Morocco | Spain | Gautret et al, 2020 |
| 22 | 2019 | Tanzania | Italy | 20191210.6832827 |
| 23 | 2019 | Philippines | Norway | 20190516.6471701 |

^1^ reference indicates ProMed-Mail archive numbers or publications as follows

Carrara, P., Parola, P., Brouqui, P., & Gautret, P. (2013). Imported human rabies cases worldwide, 1990–2012. PLoS neglected tropical diseases, 7(5), e2209.

Gautret, P., Diaz-Menendez, M., Goorhuis, A., Wallace, R. M., Msimang, V., Blanton, J., ... & Grobusch, M. P. (2020). Epidemiology of rabies cases among international travellers, 2013–2019: A retrospective analysis of published reports. Travel Medicine and Infectious Disease, 36, 101766.

Table S2. List of African trypanosomiasis imported events included in this study (from 2010 to 2019)

| ID | Year | Source country | Imported country | Reference ^1^ |
| --- | --- | --- | --- | --- |
| 1 | 2010 | Zambia | United Kingdom | 20101022.3833000 |
| 2 | 2010 | Zambia | United States | 20100915.3338000 |
| 3 | 2010 | Zambia | South Africa | 20101111.4093000 |
| 4 | 2012 | Zambia | Germany | Richter et al 2012 |
| 5 | 2012 | Tanzania | Israel | Meltzer et al 2012 |
| 6 | 2012 | Tanzania | Sweden | 20121222.1465181 |
| 7 | 2012 | Zimbabwe | United States | 20121013.1341050 |
| 8 | 2013 | Zambia | Argentina | Pale et al, 2013 |
| 9 | 2013 | Zimbabwe | Canada | Pasternak et al, 2013 |
| 10 | 2014 | Gabon | China | Zhou et al, 2018 |
| 11 | 2015 | Zambia | Canada | 20150904.3625005 |
| 12 | 2015 | Tanzania | Spain | Gomez-Junyent et al 2017 |
| 13 | 2016 | Zambia | United States | 20161226.4723752 |
| 14 | 2017 | Gabon | China | Zhou et al, 2018 |
| 15 | 2017 | Tanzania | China | Zhou et al, 2018 |
| 16 | 2017 | Zambia | Germany | 20170817.5255709 |
| 17 | 2017 | Tanzania | Netherlands | 20170518.5042860 |
| 18 | 2018 | Malawi | Netherlands | 20181124.6162718 |
| 19 | 2018 | Malawi | South Africa | 20181224.6221997 |
| 20 | 2018 | Zambia | South Africa | 20181224.6221997 |
| 21 | 2019 | Malawi | South Africa | 20191016.6730249 |
| 22 | 2019 | Uganda | South Africa | 20191002.6704899 |
| 23 | 2019 | Zambia | South Africa | 20190320.6377064 |

^1^ reference indicates ProMed-Mail archive numbers or publications as follows

Richter J, Göbels S, Göbel T, Westenfeld R, Müller-Stöver I and Häussinger D (2012) A returning traveller with fever, facial swelling, and skin lesions. BMJ 344, e2092.

Meltzer E, Leshem E, Steinlauf S, Michaeli S, Sidi Y and Schwartz E (2012) Human African Trypanosomiasis in a traveler: diagnostic pitfalls. American Journal of Tropical Medicine and Hygiene 87, 264–266.

Pale CA and Vigna L (2013) Images in clinical medicine. African Trypanosomiasis in Argentina. New England Journal of Medicine 369, 763.

Pasternak J, Wey SB, Silveira PA and Camargo TZ (2013) An African visitor in Brazil. Einstein (Sao Paulo) 11, 261–262

Zhou, X. N., Qian, M. B., Priotto, G., Franco, J. R., & Guo, J. G. (2018). Tackling imported tropical diseases in China. Emerging Microbes & Infections, 9.

Gomez-Junyent, J., Pinazo, M. J., Castro, P., Fernandez, S., Mas, J., Chaguaceda, C., ... & Muñoz, J. (2017). Human African Trypanosomiasis in a Spanish traveler returning from Tanzania.
